# RNA-seq data from whole rice grains of pigmented and non-pigmented Malaysian rice varieties

**DOI:** 10.1016/j.dib.2020.105432

**Published:** 2020-03-16

**Authors:** Rabiatul-Adawiah Zainal-Abidin, Zamri Zainal, Zeti-Azura Mohamed-Hussein, Norliza Abu-Bakar, Mohd Shahril Firdaus Ab Razak, Sanimah Simoh, Yun Shin Sew

**Affiliations:** a Malaysian Agricultural Research & Development Institute (MARDI), 43300 Serdang, Selangor, Malaysia; b Institute of Systems Biology (INBIOSIS), Universiti Kebangsaan Malaysia, 43600 UKM Bangi, Selangor, Malaysia; c Centre for Frontier Sciences, Faculty of Science & Technology, Universiti Kebangsaan Malaysia, 43600 UKM Bangi*,* Selangor, Malaysia

**Keywords:** Rice grain, Pigmented rice, Transcriptome, Nutritional trait, Quality trait

## Abstract

Pigmented rice is enriched with antioxidants, macro- and micronutrients. A comprehensive investigation of the gene expression patterns among the pigmented rice varieties would help to understand the cellular mechanism and biological processes of rice grain pigmentation. Hence, we performed RNA sequencing and analysis on the whole grain of dehusked mature seeds of selected six Malaysian rice varieties with varying grain pigmentations. These varieties were black rice (BALI and Pulut Hitam 9), red rice (MRM16 and MRQ100) and white rice (MR297 and MRQ76). Illumina HiSeq™ 4000 sequencer was used to generate total raw nucleotides of approximately 53 Gb in size. From 353,937,212 total paired-end raw reads, 340,131,496 total clean reads were obtained. The raw reads were deposited into European Nucleotide Archive (ENA) database and can be accessed via accession number PRJEB34340. This dataset allows us to identify and profile all expressed genes with functions related to nutritional traits (i.e. antioxidants, folate and amylose content) and quality trait (i.e. aroma) across both pigmented and non-pigmented rice varieties. In addition, the transcriptome data obtained will be valuable for discovery of potential gene markers and functional SNPs related to functional traits to assist in rice breeding programme.

Specifications table

**Table:** 

Subject	Agricultural and Biological Sciences
Specific subject area	Plant transcriptomics
Type of data	Table, text file
How data were acquired	Illumina HiSeq™ 4000 sequencing platform
Data format	Raw (FASTQ)
Parameters for data collection	Mature rice seeds of 6 rice varieties with varying grain pigmentations namely black rice (BALI and Pulut Hitam 9), red rice (MRM16 and MRQ100) and white rice (MR297 and MRQ76) were collected from MARDI rice field plots. BALI is a landrace rice variety while PH9, MRM16, MRQ100, MR297 and MRQ76 are modern and cultivated rice varieties. BALI, PH9, MRM16 and MRQ100 were chosen due to their high antioxidant properties [1]. MRQ76 is an aromatic rice variety [2] while MR297 has shown to have high micronutrient content [1]. The seeds were dehusked and the whole rice grains were used for total RNA extraction, cDNA library preparation and sequencing.
Description of data collection	RNAseq dataset was collected from paired-end sequencing of rice cDNA libraries using Illumina HiSeq4000™ platform with 2 × 150 bp reads. The raw reads were recorded in a FASTQ file. Raw reads were filtered to remove reads containing adapter or reads of low quality, and clean reads were mapped to reference genome of *Oryza sativa* japonica cv. Nipponbare. Total mapped reads and number of transcripts were estimated from transcript assembly with a threshold of FPKM ≥ 0.1.
Data source location	City/Town/Region: Serdang, Selangor Country: Malaysia Latitude and longitude (and GPS coordinates) for collected samples/data:] 2.9885871″N 101.697955417″E
Data accessibility	The raw paired-end transcriptome sequence reads from BALI, PH9, MRM16, MRQ100, MR297 and MRQ76 were deposited in the ENA database (www.ebi.ac.uk/ena) under the accession number PRJEB34340. Direct URL to data: https://www.ebi.ac.uk/ena/browser/view/PRJEB34340

## Value of the data

• These RNA-seq data obtained from the selected 6 rice varieties which represent the first complete set of transcriptome data generated from rice varieties with varying grain pigmentations (black, red and white).
• This dataset allows us to discover functional genes related to rice grain pigmentation, nutritional and aromatic properties.
• These data permit comparative transcriptomics between pigmented and non-pigmented rice varieties. Differential gene expression profiles between varieties could help in understanding of molecular mechanisms and biological processes that responsible for certain valuable rice trait.
• These RNAseq data together with rice genomic data are important for identification of functional markers such as single nucleotide polymorphisms (SNPs) and microsatellites related to nutritional and quality traits for future rice genetic improvement research.

## 1 Data description

The dataset in this article is RNA-seq raw reads for dehusked whole rice grains obtained from mature seeds of four pigmented (BALI, Pulut Hitam 9, MRM16 and MRQ100) and two non-pigmented (MR297 and MRQ76) rice varieties. Raw data obtained from Illumina HiSeq™ 4000 sequencer were deposited as FASTQ format in ENA database (accession number: PRJEB34340). The accession number for individual rice variety in ENA database were presented as ENA run primary accession in Table 1. Analyses of sequencing data from each rice variety e.g. raw and clean reads, raw and clean nucleotide were performed as shown in Table 2. The quality of clean reads were assessed and the percentage of high quality clean reads were obtained. By mapping clean reads to *Oryza sativa* japonica cv. Nipponbare reference genome, the number of mapped reads were estimated (Table 3). *Oryza sativa* japonica cv. Nipponbare genome was used for clean reads mapping due to it is a well-assembled and annotated genome. Although a few indica rice cultivars have been sequenced however those genomes were not well-annotated [3]. Additionally, transcript assembly to reference genome with a threshold of FPKM ≥ 0.1 predicted the number of transcripts for each rice variety as listed in Table 3.

**Table 1:** List of accession number of individual pigmented and non-pigmented rice transcriptome in ENA database.

Rice variety	Phenotype	ENA studies primary accession	ENA run primary accession
BALI	Pigmented (Black)	PRJEB34340	ERR3515585
Pulut Hitam 9	Pigmented (Black)	PRJEB34340	ERR3515586
MRM16	Pigmented (Red)	PRJEB34340	ERR3515587
MRQ100	Pigmented (Red)	PRJEB34340	ERR3515588
MR297	Non-pigmented (White)	PRJEB34340	ERR3515589
MRQ76	Non-pigmented (White)	PRJEB34340	ERR3515590

**Table 2:** Statistics of sequencing data of individual pigmented and non-pigmented rice variety.

Rice variety	Phenotype	Raw reads (paired-end)	Raw nucleotides (bp)	Clean reads (paired-end)	Clean nucleotides (bp)
BALI	Pigmented (Black)	53,901,374	8085,206,100	52,008,296	52,008,296
Pulut Hitam 9	Pigmented (Black)	63,166,848	9475,027,200	61,139,386	61,139,386
MRM16	Pigmented (Red)	61,143,304	9171,495,600	58,422,974	58,422,974
MRQ100	Pigmented (Red)	47,999,632	7199,944,800	45,725,970	45,725,970
MR297	Non-pigmented (White)	73,151,820	10,972,773,000	71,132,050	71,132,050
MRQ76	Non-pigmented (White)	54,574,234	8186,134,100	51,702,820	51,702,820
Total		353,937,212	53,090,580,800 ∼53 Gb	340,131,496	51,019,724,400 ∼51 Gb

**Table 3:** Statistics of reads mapping and transcripts assembly for each pigmented and non-pigmented rice variety.

Rice variety	High-quality reads (paired-end)	Percentage of high quality reads (%)	Mapped reads	Percentages of mapped reads (%)	Number of transcripts
BALI	52,008,296	98.19	41,648,421	80.08	24,307
Pulut Hitam 9	61,139,386	99.40	49,891,537	81.6	26,223
MRM16	58,422,974	99.31	46,768,318	80.05	25,066
MRQ100	45,725,970	94.43	34,637,723	75.75	25,123
MR297	71,132,050	99.26	55,444,321	77.95	25,416
MRQ76	51,702,820	99.36	40,626,254	78.58	25,092
Total	340,131,496		269,016,574		

## 2 Experimental design, materials, and methods

### 2.1 Plant materials, total RNA extraction and quality assessment of total RNA

Mature seeds of each pigmented and non-pigmented rice variety were obtained in the field plots at MARDI Seberang Perai, Penang, Malaysia. The seeds were dehusked and the whole rice grain tissue (three plants of each variety) were ground into fine powder using liquid nitrogen. Total RNA extraction was performed using MTL method [4] with modifications. NanoDrop ND-1000 (Thermo Scientific, Waltham, MA, USA) ultraviolet spectrophotometer was used to evaluate the isolated total RNA quantity and 1% (w/v) agarose gel electrophoresis was used to observe for the RNA degradation and contamination.

### 2.2 Library preparation and transcriptome sequencing

High-quality total RNA samples with RIN values ≥ 6.5 were subjected to isolation of messenger RNAs using oligo(dT) beads and cDNA synthesis was performed using random hexamers and SuperScript II Reverse Transcriptase (Invitrogen, USA) according to manufacturers' instructions. After that, second-strand synthesis by nick-translation was carried out using a custom second-strand synthesis buffer (Illumina) added with dNTPs, RNase H and *Escherichia coli* polymerase I. The cDNA library was then constructed after a round of purification, terminal repair, A-tailing, ligation of sequencing adapters, size selection and PCR enrichment. The cDNA library concentration was quantified using a Qubit 2.0 fluorometer (Life Technologies, USA), and then diluted to 1 ng/µl before checking insert size on an Agilent 2100 bioanalyzer (Agilent Technologies, USA). Paired-end sequencing was performed on the cDNA fragments from the resulting libraries using Illumina HiSeq4000™ platform with read length of 150 bp at each end.

### 2.3 Repository and processing of RNA-seq raw data

The sequencing raw reads were deposited into European Nucleotide Archive (ENA) (https://www.ebi.ac.uk/ena) with an accession number of PRJEB34340. Table 1 shows the ENA Run Primary accession numbers of individual pigmented and non-pigmented rice transcriptome in ENA database. The raw reads were subsequently filtered using Trimmomatic version 0.36 [5] to remove the adapter sequences, contamination and low-quality reads. Table 2 shows the statistics of raw and clean reads of individual rice transcriptome after sequence processing and analysis.

### 2.4 Reads mapping, transcripts assembly and gene expression analysis

The clean reads were mapped to the reference genome of *Oryza sativa* japonica cv. Nipponbare. Bowtie2 version 2.3.0 was used to index the reference genome, while TopHat2 version 2.0.12 [6] was used to map the clean reads onto the reference genome. The default parameters were used for the above analyses. HTSeq version 0.6.1 [7] was used to estimate the Fragments Per Kilobase of transcript per Million mapped reads (FPKMs) that were mapped to each rice gene. A threshold of FPKM ≥ 0.1 was used to determine the significance of gene expression. Cufflinks version 2.1.1 [8] was used to combine and assemble the mapped reads into the transcript. The number of mapped reads, percentage of mapped reads and number of transcripts are shown in Table 3. These sequences and information will be used for further downstream analyses such as differential expressed genes, genes co-expression network and SNPs calling.

## Appendix Supplementary materials

Image, application 1
